# A miR-JL-5 inhibitor modulates the proliferation of *Nosema cerana*e in *Apis cerana*

**DOI:** 10.3389/finsc.2025.1583941

**Published:** 2025-06-04

**Authors:** Xu Han, Jin Hua Xiao, Wei Yu Yan, Xu Jiang He, Zhi Jiang Zeng

**Affiliations:** ^1^ Honeybee Research Institute, Jiangxi Agricultural University, Nanchang, China; ^2^ Department of Animal Science, Jiangxi Biotech Vocational College, Nanchang, Jiangxi, China; ^3^ Jiangxi Key Laboratory of Honeybee Biology and Bee Keeping, Nanchang, Jiangxi, China

**Keywords:** honeybees, microsporidia, microRNA inhibitor, RNA-seq, target gene expression

## Abstract

**Background and aims:**

*Apis cerana* is the native host of *Nosema ceranae* (*N. ceranae*). Previous studies reported that *N. ceranae* was more harmful to the new host (*Apis mellifera*) than the original host that had spread globally and become one of the factors implicated in honeybee colony collapses. Therefore, it was essential to study the relationship between *Apis cerana* and *N. ceranae* for the prevention and control of the disease.

**Methods:**

In order to effectively block the regulation of spore proliferation in honeybees, we designed a miR-JL-5 inhibitor. In this study, infected bees (*Apis cerana*) were fed sugar water with *N. ceranae* miR-JL-5 inhibitor in incubators using RNA interference technology. The spores load and the expression of microRNA, DEmRNA, and target gene among miRNA inhibitor group, infection group, and miRNA scramble group were compared. Both the biological functions of miR-JL-5 and the target gene of miRNAJL-5, were verified using RNA interference.

**Results:**

Our results showed that expression levels of JL-5 and the spore load in the miRNA inhibitor group were significantly lower than those in the infection and miRNA-scramble groups. We predicted that 847 honeybee genes and 133 N*. ceranae* genes can be targeted by the miR-JL-5. Whole transcriptome sequencing results showed that a total of 5 honeybee DEmRNAs, and 0 N*. ceranae* DEmRNA were identified in miRNA inhibitor group, infection group, and miRNA scramble group, in which only the honeybee mucin-19-like gene was the target gene by miR-JL-5.

**Conclusion:**

These findings reveal that feeding miR-JL-5 inhibitor could reduce *N. ceranae* levels by altering the expression of miR-JL-5 and related target gene (honeybee mucin-19-like gene). Our results provide insights into role that microRNA regulates the proliferation of *N. ceranae* in *Apis cerana*.

## Introduction

Microsporidia are eukaryotic organisms capable of infecting vertebrates and invertebrates. They are obligate intracellular parasites and cause adverse effects to infected animals. Honeybees are vital pollinators in terrestrial ecosystems which are important for the development and maintenance of natural ecosystems and agriculture, and they are commonly infected with *Nosema* spores.

In 1996, *N. ceranae* was initially isolated and identified from a sample of *A. cerana* located near Beijing (1). As an obligate intracellular fungal parasite, *N. ceranae* mainly infects the midgut tissue of adult worker honeybees, causing serious chronic disease and a range of adverse effects on the physiology, behavior, and immune response of honeybees (1, 2). With the rise of globalization, *N. ceranae* has disseminated extensively across the world and contributed to the collapse of honeybee colonies (3, 4). The spore’s special structure consists of a dense protective layer surrounding the nucleus and cytoplasm, which enables their survival in the external environment for extended periods, even up to several years (5).

In 1993, Victor Ambros and Gary Ruvkun discovered microRNAs and its role in transcriptional gene regulation (6, 7). Studies have shown that in organisms, a single miRNA can regulate multiple genes, or multiple miRNAs can regulate the same gene (8). The *N. ceranae* is known to regulate the overall gene expression of honeybees during infection (9). Shao et al. discovered that parasite miRNAs were involved in both self-regulation during spore proliferation and interference with host gene expression (10). This finding shed light on the complex relationship between parasites and their hosts and highlighted the potential role of miRNA in modulating this interaction.

We engineered a sequence-specific inhibitor targeting NC-miR-JL-5 (a conserved microRNA in *N. ceranae*) for feeding infected honeybees, predicted and analyzed the differentially expressed mRNAs (DEmRNAs) in host and microsporidians targeted by *N. ceranae* miR-JL-5, and assessed its role in host-parasite interactions at both miRNA and mRNA levels.

## Materials and methods

### Fungal spores and honeybees

The Percoll discontinuous gradient centrifugation protocol was followed in this work (11).

Honeybees infected with *N. ceranae* were collected from the honeybee research institute of Jiangxi Agriculture University. Midguts of these honeybees were removed and crushed in sterile water. The suspension was centrifuged at 3,000×g for 5 minutes at 4°C and the supernatant discarded. The re-suspended pellet was further purified on a discontinuous Percoll (Solarbio, Beijing, China) gradient consisting of 1 ml each of 25%, 50%, 75% and 100% Percoll solution. The spore suspension was overlaid onto the gradient and centrifuged at 8,000×g for 20 minutes at 4°C. The supernatant was discarded and the spore pellet re-suspension in 1 mL distilled sterile water. Repeat the above steps of purification and suspension at least twice. The final suspension was centrifuged at 10,000×g for 5 minutes at 4°C and the supernatant discarded and the spore pellet was washed by centrifugation in distilled sterile water at least twice. Purified spores subsequently archived at the honeybee research institute of Jiangxi Agriculture University. *A. cerana* workers were collected from three different colonies reared at Honeybee Research Institute, Jiangxi Agriculture University. The selected colonies had no outbreaks of diseases, and were visually free of *Varroa* mite parasites. Three combs containing a large number of capped-brood cells were obtained from different colonies and placed in a climatic incubator (AIKANE-DHK 150, Shanghai, China), where the temperature and humidity were maintained at 30°C and 60%, respectively. All experimental honeybees were collected within 6 h of emergence and placed in plastic cups (98mmticcet)with holes.

### Microscopic watch and PCR verification of *N. ceranae* spores

The prepared spores of *N. ceranae* were identified and observed using an optical microscope (Nikon Eclipse Ci, Tokyo, Japan). Total RNA of spores was isolated and utilized as templates for reverse transcription. Subsequently, the cDNA generated was used as templates for PCR amplification using specific primers for *N. ceranae* and *N. apis* as previously described (12, 13). The amplified products were identified via 1.5% agarose gel electrophoresis (AGE). The positive control utilized *N. ceranae*. The negative control utilized sterile water.

### *N. ceranae* miR-JL-5 inhibitor

A study by Huang et al. identified six miRNAs in *N. ceranae* (14). Further analysis revealed that five of these miRNAs exhibit dual targeting functions (15). Based on the mature sequences of the aforementioned miRNAs, we designed corresponding miRNA inhibitors and identified the miR-JL-5 inhibitor as the most effective candidate through *N. ceranae* infection experiments, prioritizing it for further mechanistic and therapeutic research.

Based on the mature sequence of miR-JL-5 from *N. ceranae*, which involved in self-regulation during the proliferation. We designed and synthesized an inhibitor targeting miR-JL-5 to interfere with processes involved in microsporidia infection of honeybees (**Table 1**). Simultaneously, we created a miRNA that targeted no specific genes for use as a randomized control group.

**Table 1:** miRNA and miRNA inhibitor of *N. ceranae*.

RNAs	Type	Position	Mature sequence
JL-5	miRNA	Intergenic	uauaugucuaaucugguuuuugga
JL-5 inhibitor	ssRNA	Exogenous	uccaaaaaccagauuagacauaua

### Parasite infection and miRNA feeding

A total of 180 newly emerged worker honeybees (*A. cerana*) were randomly divided into 3 groups, with 3 replications per group and 20 honeybees per replication. Firstly, 60 newly emerged honeybee workers were selected for the infection group were inoculated with 10^5^ N*. ceranae* spores and fed with 50% sugar water. Secondly, 60 honeybees were chosen for the miRNA inhibitor group, where they were exposed to 10^5^ N*. ceranae* spores and fed with 50% sugar water containing 20 μg/mL of the miRNA inhibitor. Finally, a third group of 60 honeybees were chosen for the miRNA-scramble group. These honeybees were then infected with 10^5^ spores and fed with 50% sugar water containing 20 μ g/mL of non-matching (scrambled) miRNA.

To ensure the efficiency of miRNA feeding, we referred to the method proposed by Maori et al. (16). Therefore, except for infection group, 120 μg of the miRNA preparation was added to each inhibitor groups or scramble group daily in 6 mL of 50% sucrose solution. The miRNA preparations were added to the sucrose solutions to a final concentration of 20 μg/ml and miRNA preparations were supplemented daily for another seven days.

### Sample collection and spores counting

Fifteen honeybee workers were collected from each group on the 7^th^ day after infection. Total RNA was extracted from the midgut tissues of five honeybees from each repeating group using the RNA Extraction Kit (TaKaRa Company, Dalian, China). Equal amounts of RNA from each biological replicate group were then pooled for mRNA sequencing. Next, the rest of the honeybees from each group were collected as only one pooled sample and individual mid-gut tissues were homogenized to count the spore loads using an optical microscope (Nikon Eclipse Ci, 400×magnification) with the Neubauer Improved Hemocytometer (0.1 mm depth). Data analyses were performed using One-way ANOVA test.

### Target gene prediction

The software miRanda (3.3a) was used to predict the target genes of miRNA-JL5 in the genomes of A. cerana and *N. ceranae*. The parameters for Miranda (v3.3a) were -sc 140 -en 10 -scale 4 -strict -out.

### RNA-seq and validation of DEmRNA by RT-qPCR

The mRNA was sequenced with Illumina Hiseq2000, and the raw counts were normalized. The clean reads were first mapped to the reference genome of *A. cerana* (assembly ASM1110058v1) to obtain host-derived data, and the unmapped clean reads were further mapped to the *N. ceranae* reference genome (assembly Ncer 3.0) to obtain microsporidian-derived data. Gene expression levels were compared between the *N. ceranae* infection group, miR-JL-5 inhibitor group, and miRNA scramble group. The edgeR package was used to identify significantly expressed genes (|log2FoldChange|>0.6, adjusted p<0.05). To validate the accuracy of the transcriptome datasets employed in this study, according to the targeted binding relationship predicted by the software, the host DEmRNA (mucin-19-like gene, LOC108003100) both in the miRNA inhibitor group vs. the infection group and the miRNA inhibitor group vs. the miRNA-scramble group, the microsporidian miR-JL-5, were selected for RT-qPCR. Specific forward and reverse primers for the DEmRNAs and actin were designed using Primer Premier 5 (**Supplementary Table S1**). Total RNA was reverse transcribed into cDNA using oligo(dT) primers, and the cDNA was subsequently used as a template for qPCR analysis. The qPCR was performed on a CFX Connect™ Real-Time PCR System (Bio-Rad Laboratories, Shanghai, China) using Magic SYBR Mixture (CWBIO, Jiangsu, China). The thermal cycling conditions were as follows: initial denaturation at 95°C for 1 min, followed by 40 cycles of 95°C for 15 s, 55°C for 30 s, and 72°C for 45 s. Relative gene expression levels were calculated using the 2^−ΔΔCt^ method. Each experiment was performed in triplicate using three independent biological replicates.

## Results

### Verification of infection of *A. cerana* worker by *N. ceranae*

**Figure 1:**
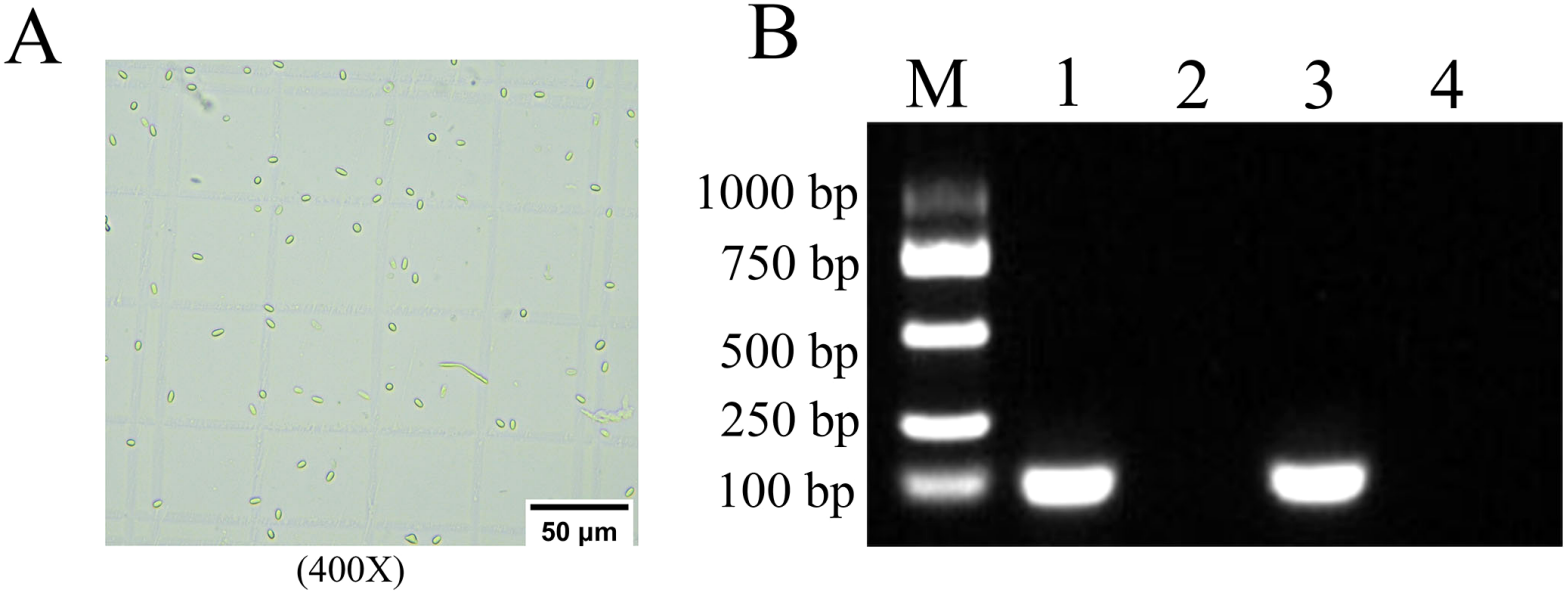
Microscopic view and PCR verification of *N. ceranae* Spores. **(A)** Microscopic detection, 400 times magnification. **(B)** AGE for PCR amplified fragments, Lane M: DNA marker, Lane 1: The cDNA of the purified spores and Specific primers for *N. ceranae*, Lane 2: The cDNA of the purified spores and Specific primers for *N. apis*, Lane 3: The DNA for *N. ceranae* and Specific primers for *N. ceranae* and *N. apis* (Positive control), Lane 4: Sterile water and Specific primers for *N. ceranae* and *N. apis* (Negative control).

Under an optical microscope (Nikon Eclipse Ci, 400×magnification), oval and highly refractive dispersed spores with the characteristics of Microsporidia were observed (**Figure 1A**). Furthermore, agarose gel electrophoresis (AGE) indicated that the expected fragment (approximately 100 bp) was amplified from the purified spores with specific primers for *N. ceranae*, *which was of the same size as the positive control group*, while no signal band was detected using specific primers for *N. apis*. No amplification products were detected in the negative control group using specific primers for *N. ceranae* and *N. apis*. (**Figure 1B**). These results verified that the purified spores were indeed *N. ceranae* spores.

### The effect of miR-JL-5 inhibitor on the number of spores in honeybees

**Figure 2:**
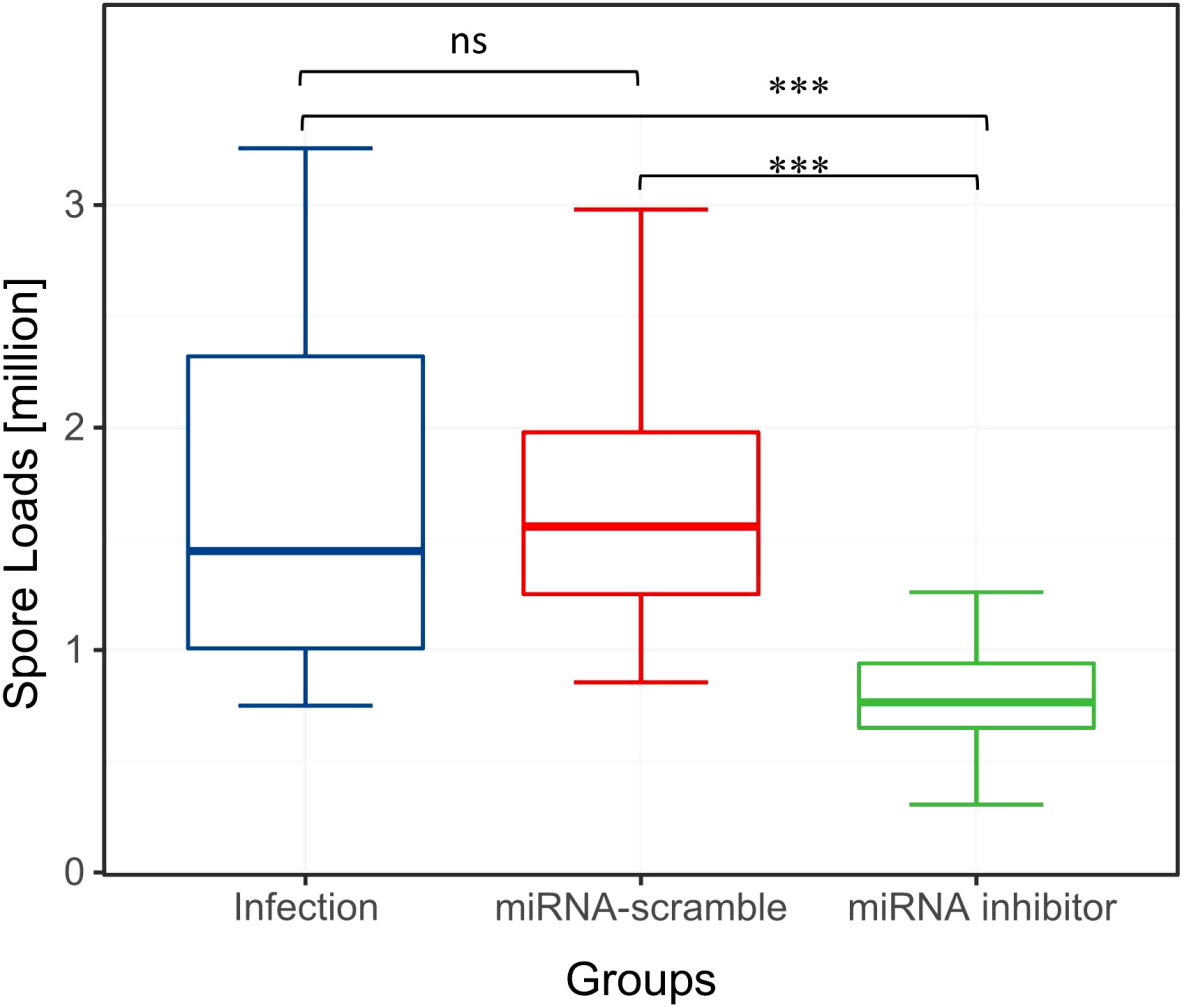
Spore loads of the three experimental groups. Overall, compared to the other two groups, the effect of miRNA inhibitor group on the spore loads was significant (ANOVA, p < 0.05). "ns" indicates "no significant difference". "***" indicates a statistically significant difference at P<0.001.

At 7 dpi, the analysis of variance (One-way Anova) on spore count data across groups revealed statistically significant between-group differences (F = 54.950, p < 0.001). As expected, the *N. ceranae* spore count in the miRNA inhibitor group was significantly lower than that in the infection and miRNA-scramble groups (**Figure 2**, p < 0.05). There was no significant difference in the number of *N. ceranae* spores between the infection group and the miRNA-scramble group (**Figure 2**, p > 0.05).

### Prediction of miR-JL-5 target genes

In total, 847 honeybee genes and 133 N*. ceranae* genes were predicted as the target genes of miR-JL-5 by miRanda (3.3a) software. The above prediction results still needed to be further verified through experiments.

### The effects of the miRNA-JL5 inhibitor on host and parasite mRNA levels

In order to further verified the target genes of the miR-JL-5 of honeybee and *N. ceranae*, we screened the predicted target genes experimentally. For honeybees or *N. ceranae*, the gene expression levels were pair-wise compared among the miRNA inhibitor, infection and miRNA-scramble groups. The significantly expressed honeybee genes and *N. ceranae* genes due to miRNA-inhibitor feeding must meet three criteria (17): (1) the genes were significantly differently expressed between the miRNA inhibitor group and the infection group; (2) the genes were significantly differently expressed between the miRNA-inhibitor group and the miRNA-scramble group; and (3) the genes were not significantly differentially expressed between the miRNA-scramble group and the infection group.

**Figure 3:**
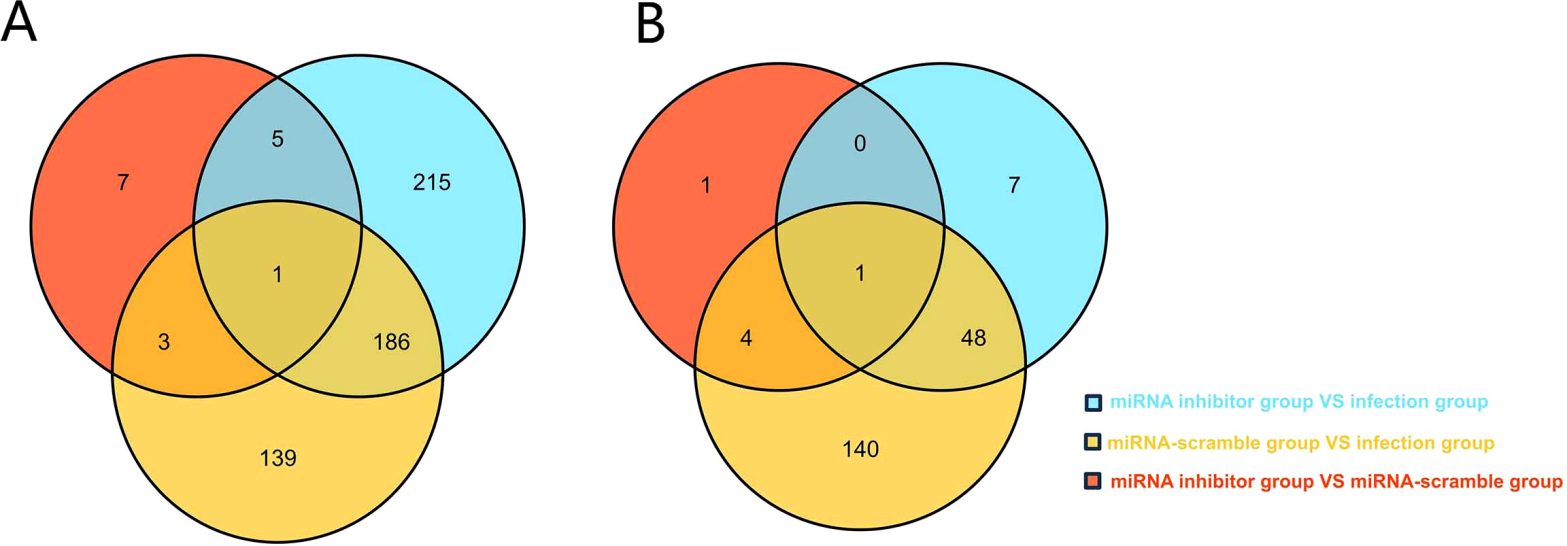
**(A)** Venn analysis of DEGs in *N. ceranae* in the miRNA inhibitor group vs. the miRNA scramble group, the miRNA inhibitor group vs. the infection group, and miRNA scramble group vs. the infection group. **(B)** Venn analysis of DEGs in *A*. *cerana* in the miRNA inhibitor group vs. the miRNA scramble group, the miRNA inhibitor group vs. the infection group, and miRNA scramble group vs. the infection group.

As shown in **Figure 3A**, there were no *N. ceranae* genes which expression met the above criteria. There were five *A. cerana* genes that met these criteria, but only the mucin-19-like gene was one of the target genes predicted computationally (**Figure 3B**).

### The effects of miR-JL-5 inhibitor on miRNA/mRNA expression levels

**Figure 4:**
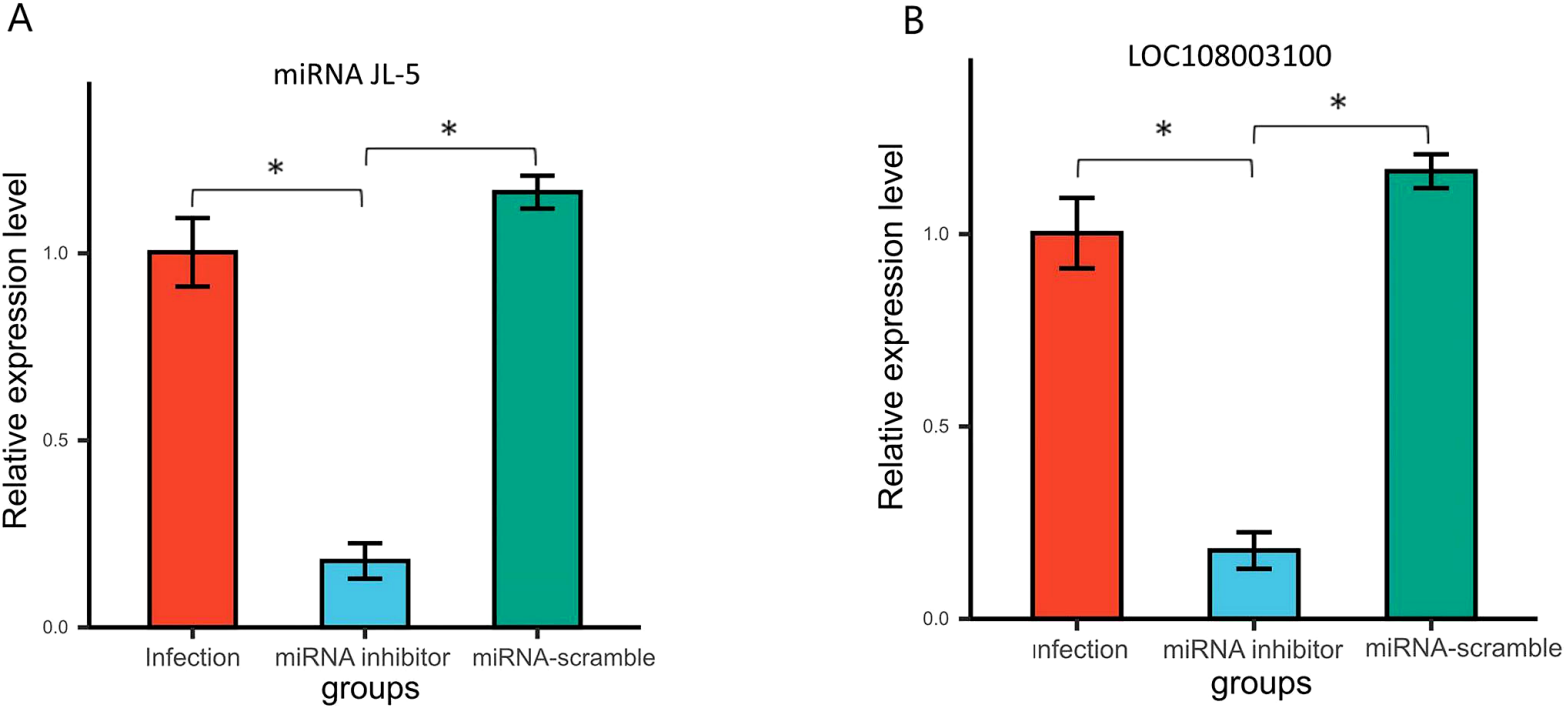
**(A)** Venn analysis of DEGs in *N. ceranae* in the miRNA inhibitor group vs. the miRNA scramble group, the miRNA inhibitor group vs. the infection group, and miRNA scramble group vs. the infection group. **(B)** Venn analysis of DEGs in *A*. *cerana* in the miRNA inhibitor group vs. the miRNA scramble group, the miRNA inhibitor group vs. the infection group, and miRNA scramble group vs. the infection group. Bars with asterisk symbol indicate statistically significant differences (p < 0.05).

To validated the *in vivo* impact of miR-JL-5 inhibitor administration on miRNA and mRNA expression in honeybees. We quantified the JL-5 and mucin-19-like gene (LOC108003100) expression in the honeybee midguts 7 dpi after administering the miRNA inhibitor using qRT-PCR. The qRT-PCR results demonstrated that the miR-JL-5 inhibitor effectively reduced the expression of JL-5 (*p* < 0.05) and increased the expression of mucin-19-like gene (*p* < 0.05). However, they remained unaffected by the control miRNA-scramble group (*p* > 0.05) (**Figure 4**).

## Discussion

MiRNAs play a crucial role in controlling gene expression and biological processes, facilitating cross-kingdom regulation between plants, animals, and microorganisms. (18, 19). Notably, despite microsporidia being among the simplest eukaryotes (20), certain miRNAs have been shown to modulate host gene expression in *N. ceranae*, highlighting a potential role for miRNAs in microsporidian infections (21, 22). In this current study, the miRNA inhibitor group showed significantly fewer spore counts than the other groups, which consistent with our experimental expectations (**Figure 2**). A total of 847 honeybee genes and 133 *Nosema* genes were predicted as miR-JL-5 target genes by the software miRanda (3.3a). The number of host genes targeted by miR-JL-5 was significantly higher than the number of parasite genes so targeted. Notably, among the experimental results analyzed in the Venn diagram, only the honeybee mucin-19-like gene met the criteria for differential gene expression, consistent with computational target predictions (**Figure 3**). It is discovered that down-regulated *N. ceranae* miR-JL-5 in *A. cerana* worker’s midgut at 7 dpi could target up-regulated mRNA (mucin-19-like gene) in *A. cerana*, while none of regulated mRNAs in microsporidian was targeted by miR-JL-5 (**Figure 4**). Perhaps the spore-forming parasites may possess a unique spore wall structure that renders them resistant to inhibitors.

The *N. ceranae* is known to alter the expression of honeybee midgut proteins to create a favorable environment for parasite development. Mucins, a category of high molecular weight, heavily glycosylated proteins with chitin binding domain, are the primary element of the mucus barrier secreted by epithelial cells for intestinal protection (23, 24). Mucin genes were regulated by host innate responses and played a significant role in defending against intestinal infections. (25). Huang et al. discovered that by feeding infected honeybees with small interfering RNA targeting the *N. ceranae* gene coding Dicer (siRNA Dicer), and the spore loads were significantly reduced, as well as the honeybee gene mucin-2-like showed significantly up regulation in the siRNA-Dicer group compared with the infection group (17). In our study, the relationship between mucin-19-like gene and miR-JL-5 was correlative. However, the miR-JL-5 treatment and the reduced *N. ceranae* level was causative. It provided novel insights into the cross-kingdom regulation of honeybee gut by spore miRNAs during microsporidian infection. Although miRNA treatments provided a promising strategy to control pathogen infection, caution is required, as improper usages could lead to dysbiosis of the gut environment and a potential pollution risk toward bee products.

In apicultural industry, chemicals such as fumagillin have been historically and widely used against *Nosema ceranae* infection (26, 27). However, these chemicals have been banned in the European Union (EU) due to its documented toxicity and environmental residues. The U.S. patent US8822426B2 “Prevention and Treatment of Nosema Disease in Bees” (28) described a way to prevention and treatment of Nosema infections in honeybees by feeding of Nosema-specific dsRNA. Afterwards, Rodríguez-García et al. (12) demonstrated that targeted suppression of tubulin *β*-3 gene expression through RNA interference (RNAi) significantly reduced *N. ceranae* spore in infected bees; He et al. (29) found that feeding bees with dsRNA targeting to SWP8 and SWP12 genes significantly reduced *N. ceranae* spore counts, boosted immunity, and extended infected honeybees’ lifespan. However, dsRNA have a short efficiency duration and require repeated dosing (30); Some insects as honeybees and beetles lack robust systemic RNAi machinery, leading to poor dsRNA absorption and limited target tissue delivery (31). Compared to the above treatments, miRNA inhibitors have many advantages. For instance, miRNA inhibitors can precisely target pathogen or host miRNAs involved in infection (32); miRNA inhibitors also have longer stability compared to dsRNAs (33). Therefore, miRNA inhibitors could serve as a promising therapeutic strategy against honeybee parasites and pathogens.

## Conclusions

This study is the first to demonstrate that miR-JL-5, produced by *N. cerana* spores, plays a crucial role in regulating the expression of the honeybee intestinal mucin-19-like gene during the infection process. Furthermore, the findings indicate that ingesting an inhibitor targeting *N. cerana* miR-JL-5 effectively enhances (renews) the honeybee midgut’s defense capacity and reduces spore levels. The results provide insights into role that microRNA regulates the proliferation of *N. ceranae* in *Apis cerana*. Future research could explore engineering symbiotic bacteria to colonize the honeybee gut, enabling sustained production of miRNA inhibitors while reducing manufacturing costs.

## Data Availability

Whole transcriptome sequencing data have been deposited in the NCBI database under the accession ID: PRJNA1227005; PRJNA1228290.

## Supplementary material

Supplementary Table 1 The primers of *N. ceranae* and *N. apis* for PCR.

Supplementary Table 2
*Apis cerana* target genes for miR-JL-5.

Supplementary Table 3 Nosema ceranae target genes for miR-JL-5.

Supplementary Table 4 The primers of mucin-19-like gene and miR-JL-5 for PCR.

Supplementary Table 5 The expression levels of JL-5 in each group at 7 dpi.

Supplementary Table 6 The expression levels of mucin-19-like gene in each group at 7 dpi.

Supplementary Table 7 DEGs for Venn analysis in *Apis cerana* in each group.

Supplementary Table 8 DEGs for Venn analysis in *Nosema ceranae* in each group.

Supplementary Table 9 Un-targeted differentially expressed genes (DEGs) from RNA-Seq of *Apis cerana.*

Supplementary Table 10 Un-targeted differentially expressed genes (DEGs) from RNA-Seq of *Nosema ceranae.*
